# The Initial Clinical and Electrophysiological Characteristics of Different Subtypes of Guillain–Barré Syndrome Diagnosed Based on Serial Electrophysiological Examinations

**DOI:** 10.1002/brb3.70068

**Published:** 2024-09-30

**Authors:** Shuo Yang, Na Chen, Lei Zhang, Ying Wang, Lin Chen, Fan Jian, Zaiqiang Zhang, Yilong Wang, Hua Pan

**Affiliations:** ^1^ Department of Neurology, Beijing Tiantan Hospital Capital Medical University Beijing China; ^2^ National Center for Neurological Diseases Beijing China; ^3^ Chinese Institute for Brain Research Beijing China; ^4^ Advanced Innovation Center for Human Brain Protection Capital Medical University Beijing China; ^5^ China National Clinical Research Center for Neurological Diseases Beijing China; ^6^ Beijing Laboratory of Oral Health Capital Medical University Beijing China; ^7^ Beijing Municipal Key Laboratory of Clinical Epidemiology Beijing China

**Keywords:** axonal degeneration, demyelination, electrophysiology, Guillain–Barré syndrome, reversible conduction failure

## Abstract

**Background:**

We aimed to identify different Guillain–Barré syndrome (GBS) subtypes, demyelination, axonal degeneration, and reversible conduction failure (RCF) as early as possible by analyzing the initial clinical and electrophysiological examinations.

**Methods:**

This study retrospectively collected GBS patients between October 2018 and December 2022 at Beijing Tiantan Hospital. The diagnostic criteria for the initial electrophysiological study were based on Rajabally's criteria, and the criteria for the serial electrophysiological study were based on Uncini's criteria. All subjects underwent clinical and electrophysiological evaluations at least twice within 8 weeks.

**Results:**

A total of 47 eligible patients with GBS were included, comprising 19 acute inflammatory demyelinating polyradiculoneuropathy (AIDP), 18 axonal degenerations, and 10 RCFs. In the RCF group, 40%, 30%, and 30% patients were diagnosed as AIDP, axonal, and equivocal at the initial study, respectively. The AIDP group had significantly higher cerebrospinal fluid (CSF) protein than the RCF (123.8 [106.4, 215.1] mg/dL vs. 67.1 [36.8, 85.6] mg/dL, *p *= 0.002) and axonal degeneration (123.8 [106.4, 215.1] mg/dL vs. 60.8 [34.8, 113.0] mg/dL, *p* < 0.001) groups. The RCF group had significantly lower Hughes functional grades at admission (3 [2, 4] vs. 4 [4, 4], *p *= 0.012) and discharge (1.0 [1.0, 2.0] vs. 3.0 [2.0, 3.0], *p* < 0.001) than the axonal degeneration group and showed significantly shorter distal motor latency (DML), *F*_min_, *F*_mean_, *F*_max_, and lower *F*% than the AIDP group (*p *< 0.05).

**Discussion:**

The early identification of RCF from AIDP had relatively obvious features, including slightly elevated CSF protein levels and normal or slightly prolonged DML and F‐wave latencies, contrasting with the apparent elevation and prolongation seen in AIDP. Differentiating RCF from axonal degeneration remains challenging. One potential distinguishing factor is that the motor function in RCF tends to be better than in the latter.

## Graphical abstract

Our study aimed to identify reversible conduction failure (RCF) early in patients with Guillain–Barré syndrome based on initial clinical and electrophysiological examinations. The early identification of RCF from acute inflammatory demyelinating polyradiculoneuropathy (AIDP) exhibits relatively distinct features, including protein levels in cerebrospinal fluid, distal motor latency, and F‐wave latencies. However, differentiating RCF from axonal degeneration remains challenging, with the Hughes functional grade potentially serving as a distinguishing factor.

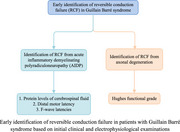

**Abbreviations**
AIDP: acute inflammatory demyelinating polyradiculoneuropathy
AMAN: acute motor axonal neuropathy
AMSAN: acute motor sensory axonal neuropathy
CB: conduction block
CMAP: compound muscle action potential
CSF: cerebrospinal fluid
DML: distal motor latency
*F*%: F‐wave persistence
*F*_max_
: maximum F‐wave latency
*F*_mean_
: mean F‐wave latency
*F*_min_
: minimum F‐wave latency
GBS: Guillain–Barré syndrome
RCF: reversible conduction failure
SNAP: sensory nerve action potential
WBC: white blood cell

## 1 Introduction

Since the first description of the axonal form of Guillain–Barré syndrome (GBS) by Feasby et al. (1986), the subsequent classification of GBS has mainly transformed to acute inflammatory demyelinating polyradiculoneuropathy (AIDP), acute motor axonal neuropathy (AMAN), and acute motor sensory axonal neuropathy (AMSAN) (Feasby et al. 1993; Griffin et al. 1996; Hughes et al. 1999; McKhann et al. 1993).

Ho et al. (1997) reported a 64‐year‐old woman who was diagnosed as AMAN and improved quickly following plasmapheresis. This process indicated the reversible potential of some AMAN patients. Kuwabara, Asahina, et al. (1998) and Kuwabara, Yuki, et al. (1998) proposed the term reversible conduction failure (RCF) in their articles. RCF can promptly recover after immune‐modulated treatment, which is due to injury located in nodal membrane of the axon or through detachment of terminal myelin loops (Kuwabara, Asahina, et al. 1998; van Doorn et al. 2023). The put forward of RCF makes the differential diagnosis of GBS subtypes more complicated.

Early recognition of different subtypes is important for determining the intervention and prognosis of these patients. If the conduction failure of axonal dysfunction was not resolved rapidly, they would progress to secondary Wallerian‐like axonal degeneration. Currently, RCF can only be detected by serial nerve conduction studies. How can we distinguish the conduction failure as soon as possible? This study tries to analyze the initial clinical and electrophysiological characteristics of different subtypes of GBS diagnosed based on serial electrophysiological examinations. We hope that these features can provide help for the early identification of different subtypes of GBS.

## 2 Methods

### 2.1 Subjects

This study retrospectively collected GBS patients admitted to Beijing Tiantan Hospital between October 2018 and December 2022. The diagnosis of AIDP, axonal degeneration, and RCF conformed to the criteria defined by Uncini based on serial examinations (Uncini et al. 2017). The diagnostic criteria for the initial electrophysiological study were based on Rajabally's criteria (Rajabally et al. 2015). All subjects underwent initial clinical and electrophysiological evaluation within 14 days from the disease onset. The second evaluation needs to be conducted within 8 weeks from the onset. This study was approved by the ethical board.

### 2.2 Clinical and Laboratory Evaluations

Clinical measurements we included in this study consisted of four limb tendon reflex and Hughes functional grade. Laboratory measurements included white blood cell (WBC) and protein of cerebrospinal fluid (CSF), and antibodies against gangliosides, including IgG, IgM against GM1, GD1a, GD1b, GT1a, GT1b, and GQ1b of CSF and blood.

### 2.3 Electrophysiological Evaluations

We collected the following information from the electrophysiological studies: motor nerve conduction studies in median, ulnar, tibial, and fibular nerves of all subjects. We measured amplitude and duration of compound muscle action potential (CMAP) induced by distal and proximal stimulations (dCMAP and pCMAP), conduction velocity, distal motor latency (DML), and minimum, mean, maximum F‐wave latency (*F*_min_, *F*_mean_, *F*_max_) and persistence (*F*%). The sensory nerve conduction studies included orthodromic median, ulnar, medial plantar, fibular nerves, and antidromic sural nerve. We measured amplitude and conduction velocity of sensory nerve action potential (SNAP).

All results of nerve conduction studies were compared with the reference values established and widely used in China (Pan et al. 2013; Tang 2002), which correspond closely to values reported in the United States (Kimura 2013). On the basis of the consensus criteria of the American Association of Electrodiagnostic Medicine, definite partial conduction block (CB) consists of more than 50%–60% amplitude reduction and 40%–50% area reduction with less than 30% duration increase between proximal and distal sites (Olney 1999). Criteria for abnormal temporal dispersion are more than 15% duration increase between proximal and distal sites (Kimura 2013).

### 2.4 Data Analysis

We used mean ± standard deviation (mean ± SD) and median (P25, P75) to describe numerical variables with normal and non‐normal distribution, respectively, and proportion to categorical variables. ANOVA and non‐parametric tests were used for comparison of normally and non‐normally distributed numerical variables, respectively. The Chi‐square test was used for comparison of categorical variables. *p* value <0.05 (two‐sided) was considered statistically significant.

## 3 Results

### 3.1 The Diagnostic Consistency Based on the Initial and Serial Electrophysiological Studies

**FIGURE 1:**
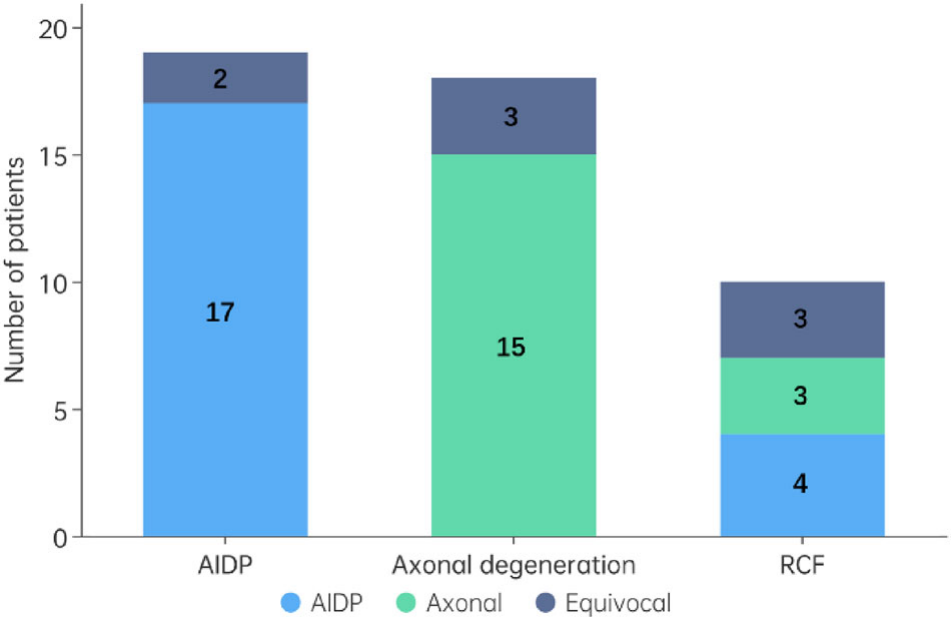
The relationship of diagnostic classifications based on the initial electrophysiological study and serial electrophysiological studies.

This study included a total of 47 eligible GBS patients, comprising 19 AIDPs, 18 axonal degenerations (12 AMAN and 6 AMSAN), and 10 RCFs (6 AMAN and 4 AMSAN), based on serial electrophysiological studies using Uncini's criteria. The diagnosis of these 47 patients was 21 AIDP, 18 axonal, and 8 equivocal based solely on the initial electrophysiological examination with Rajabally's criteria. Figure 1 shows the relationship of diagnostic classification based on the initial and serial electrophysiological studies. As we can see in Figure 1, the diagnostic consistency of initial and serial electrophysiological studies in the AIDP and axonal degeneration groups was 89% and 83%, respectively. However, in the RCF group, 40%, 30%, and 30% of patients were diagnosed as AIDP, axonal, and equivocal at the initial study, respectively.

### 3.2 Demographic, Clinical, and Laboratory Characteristics of AIDP, Axonal Degeneration, and RCF Groups

The patients’ demographics, clinical features, and laboratory measurements are presented in Table 1. Group comparisons of demographic characteristics did not reveal any significant differences. Hughes functional grade at admission, at discharge, variation from admission to discharge, and CSF protein among the three groups showed significant differences. Further pairwise comparisons between groups indicated that the AIDP group had significantly higher CSF protein levels than the RCF (*p *= 0.002) and axonal degeneration (*p *< 0.001) groups, with no significant difference in CSF collection time. The RCF group had significantly lower Hughes functional grades at admission (*p *= 0.012) and discharge (*p *< 0.001) and higher Hughes functional grade variation from admission to discharge (*p *= 0.003) than the axonal degeneration group.

**TABLE 1:** Demographic, clinical, and laboratory characteristics of different subtypes of Guillain–Barré syndrome (GBS) in the initial examination.

	AIDP (*n* = 19)	Axonal degeneration (*n* = 12 + 6)	RCF (*n* = 6 + 4)	*p*
Age, years, mean ± SD	51 ± 17	49 ± 14	48 ± 16	0.734
Sex, *n* (F/M)	9/10	11/7	5/5	0.686
Decrease of tendon reflex				
Upper limbs, *n* (%)	16 (84%)	15 (83%)	8 (80%)	0.958
Lower limbs, *n* (%)	19 (100%)	17 (94%)	8 (80%)	0.110
Hughes functional grade				
At admission, median (IQR)	4.0 (3.0, 4.0)	4.0 (4.0, 4.0)	3.0 (2.0, 4.0)	0.014
At discharge, median (IQR)	2.0 (1.8, 3.0)	3.0 (2.0, 3.0)	1.0 (1.0, 2.0)	<0.001
Variation from admission to discharge, median (IQR)	1.0 (1.0, 2.0)	1.0 (1.0, 1.0)	2.0 (1.0, 2.0)	0.005
CSF				
Collection time from onset, *d*, median (IQR)	9.0 (6.3, 12.5)	10.5 (7.0, 14.0)	8.0 (6.0, 18.0)	0.903
WBC, *n*/µL, median (IQR)	4.0 (2.0, 10.3)	2.5 (1.0, 5.3)	4.0 (2.0, 9.5)	0.367
Protein, mg/dL, median (IQR)	123.8 (106.4, 215.1)	60.8 (34.8, 113.0)	67.1 (36.8, 85.6)	<0.001
Antibodies against gangliosides				
CSF				
GM1	0/8 (0%)	3/11 (27%)	3/6 (50%)	0.090
GD1a, GD1b, GT1a, GT1b, GQ1b	0/8 (0%)	0/11 (0%)	0/6 (0%)	/
Blood				
GM1	0/8 (0%)	2/11 (18%)	3/6 (50%)	0.067
GD1a, GD1b, GT1a, GT1b, GQ1b	0/8 (0%)	0/11 (0%)	0/6 (0%)	/

Abbreviations: AIDP, acute inflammatory demyelinating polyradiculoneuropathy; CSF, cerebrospinal fluid; IQR, interquartile range; RCF, reversible conduction failure; SD, standard deviation; WBC, white blood cell.

Antibodies against gangliosides were detected in some patients with AIDP, axonal degeneration, and RCF groups. The positive rates were 0%, 27%, and 50% in CSF and 0%, 18%, and 50% in blood, respectively. All positive antibodies in this study were identified as GM1‐IgG and (or) GM1‐IgM.

### 3.3 Quantitative Comparison of Initial Electrophysiological Parameters Among AIDP, Axonal Degeneration, and RCF Groups

As shown in Table 2, the occurrence rate of CB was 47% and 60%, and abnormal temporal dispersion was 32% and 0% in the AIDP and RCF groups, respectively. Although CB and abnormal temporal dispersion did not show a significant difference between the two groups, the RCF group had significantly shorter DML in median, tibial, and fibular nerves, lower CMAP amplitude in ulnar nerve, faster CMAP velocity in median nerve, shorter *F*_min_, *F*_mean_, and *F*_max_ in tibial nerve, and lower *F*% in ulnar nerve than the AIDP group.

**TABLE 2:** Comparison of initial electrophysiological parameters between acute inflammatory demyelinating polyradiculoneuropathy (AIDP), axonal degeneration, and reversible conduction failure (RCF).

	AIDP (*n* = 19)	Axonal degeneration (*n* = 18)	RCF (*n* = 10)	*p*	RCF vs. AIDP	RCF vs. axonal degeneration	AIDP vs. axonal degeneration
					*p*	*p*	*p*
Time from onset, *d*, median (IQR)	11.0 (10.0, 14.0)	10.5 (8.0, 14.0)	10.0 (8.0, 11.5)	0.106	/	/	/
DML, ms, median (IQR)							
Median nerve	7.1 (4.6, 9.0)	3.6 (3.3, 4.0)	3.7 (3.6, 4.5)	<0.001	0.001	0.866	<0.001
Ulnar nerve	3.4 (2.9, 5.4)	2.6 (2.3, 3.1)	3.0 (2.7, 3.2)	0.001	0.277	0.377	<0.001
Tibial nerve	5.2 (4.7, 7.2)	3.9 (3.6, 4.7)	4.5 (3.9, 5.0)	<0.001	0.017	0.752	<0.001
Fibular nerve	6.0 (4.3, 7.9)	3.5 (3.1, 3.7)	3.8 (3.3, 4.0)	<0.001	0.002	1.000	<0.001
CMAP amplitude, mV, median (IQR)							
Median nerve	7.6 (5.2, 13.2)	4.9 (1.5, 9.3)	5.8 (2.6,11.5)	0.035	0.350	1.000	0.035
Ulnar nerve	11.5 (8.1, 13.0)	2.5 (0.5, 8.9)	6.4 (1.9, 10.1)	<0.001	0.047	1.000	<0.001
Tibial nerve	4.7 (2.6, 9.6)	3.2 (0.2, 8.9)	3.6 (1.0, 8.5)	0.253	/	/	/
Fibular nerve	4.0 (1.6, 6.1)	3.7 (0.9, 4.6)	3.7 (1.4, 8.2)	0.511	/	/	/
CMAP velocity 1, m/s, median (IQR)							
Median nerve	46.8 (36.2, 53.1)	53.6 (51.0, 56.0)	55.5 (51.6, 59.6)	<0.001	0.001	0.707	0.008
Ulnar nerve	51.1 (45.0, 57.6)	56.4 (52.8, 60.3)	55.7 (50.4, 62.8)	0.053	/	/	/
Tibial nerve	36.1 (28.5, 40.7)	42.6 (38.9, 46.9)	41.5 (37.3, 43.8)	0.001	0.072	1.000	0.001
Fibular nerve	37.8 (27.4, 43.4)	43.6 (41.0, 45.8)	42.8 (38.0, 45.5)	0.012	0.209	1.000	0.014
CMAP velocity 2, m/s, median (IQR)							
Median nerve	17.5 (11.8, 30.8)	38.9 (36.2, 46.8)	37.2 (31.0, 44.8)	<0.001	0.003	1.000	<0.001
Ulnar nerve	45.6 (30.8, 54.7)	52.0 (49.6, 57.0)	52.9 (47.2, 57.2)	0.040	0.189	1.000	0.058
*F*_min_, ms, median (IQR)							
Ulnar nerve	31.1 (27.2, 34.6)	26.9 (25.3, 28.4)	29.9 (26.9, 32.5)	0.010	1.000	0.237	0.009
Tibial nerve	57.9 (54.9, 68.5)	53.5 (50.3, 54.9)	52.8 (51.3, 54.5)	0.001	0.010	1.000	0.001
*F*_mean_, ms, median (IQR)							
Ulnar nerve	31.9 (28.0, 34.6)	26.9 (25.3, 28.4)	29.9 (26.9, 32.5)	0.031	1.000	0.303	0.031
Tibial nerve	58.7 (56.1, 71.6)	54.2 (51.2, 56.7)	52.5 (52.3, 55.0)	<0.001	0.011	1.000	0.001
*F*_max_, ms, median (IQR)							
Ulnar nerve	32.5 (29.3, 36.5)	28.8 (27.1, 31.0)	31.2 (29.3, 36.6)	0.031	1.000	0.332	0.030
Tibial nerve	62.1 (58.4, 73.3)	55.6 (53.1, 57.7)	55.9 (53.3, 57.8)	<0.001	0.018	1.000	0.001
*F*%, %, median (IQR)							
Ulnar nerve	100.0 (100.0, 100.0)	65.0 (43.8, 100.0)	20.0 (0.0, 91.0)	0.005	0.006	0.495	0.135
Tibial nerve	100.0 (0.0, 100.0)	85.0 (0.0, 100.0)	97.5 (20.0, 100.0)	0.469	/	/	/
Conduction block, *n* (%)	9 (47%)	0 (0%)	6 (60%)	0.001	/	<0.05	<0.05
Abnormal temporal dispersion, *n* (%)	6 (32%)	0 (0%)	0 (0%)	0.006	/	/	<0.05

*Note*: CMAP velocity 1 included velocity from elbow to wrist for median nerve, from elbow below to wrist for ulnar nerve, from popliteal fossa to ankle for tibial nerve, and from fibular head to ankle for fibular nerve. CMAP velocity 2 included velocity from wrist to palm for median nerve and from elbow above to elbow below for ulnar nerve.

Abbreviations: CMAP, compound muscle action potential; DML, distal motor latency; *F*%, F‐wave persistence; *F*_max_, maximum F‐wave latency; *F*_mean_, mean F‐wave latency; *F*_min_, minimum F‐wave latency; IQR, interquartile range.

The RCF and axonal degeneration groups showed similarities in most electrophysiological parameters, except for CB, which occurred in 60% of patients in the RCF group but not in the latter.

The AIDP group had significantly longer DML in median, ulnar, tibial, and fibular nerves, higher CMAP amplitude in median and ulnar nerves, slower CMAP velocity in median, tibial, and fibular nerves, and longer *F*_min_, *F*_mean_, and *F*_max_ in ulnar and tibial nerves than the axonal degeneration group. The occurrence rate of abnormal temporal dispersion also showed significant differences between two groups.

**FIGURE 2:**
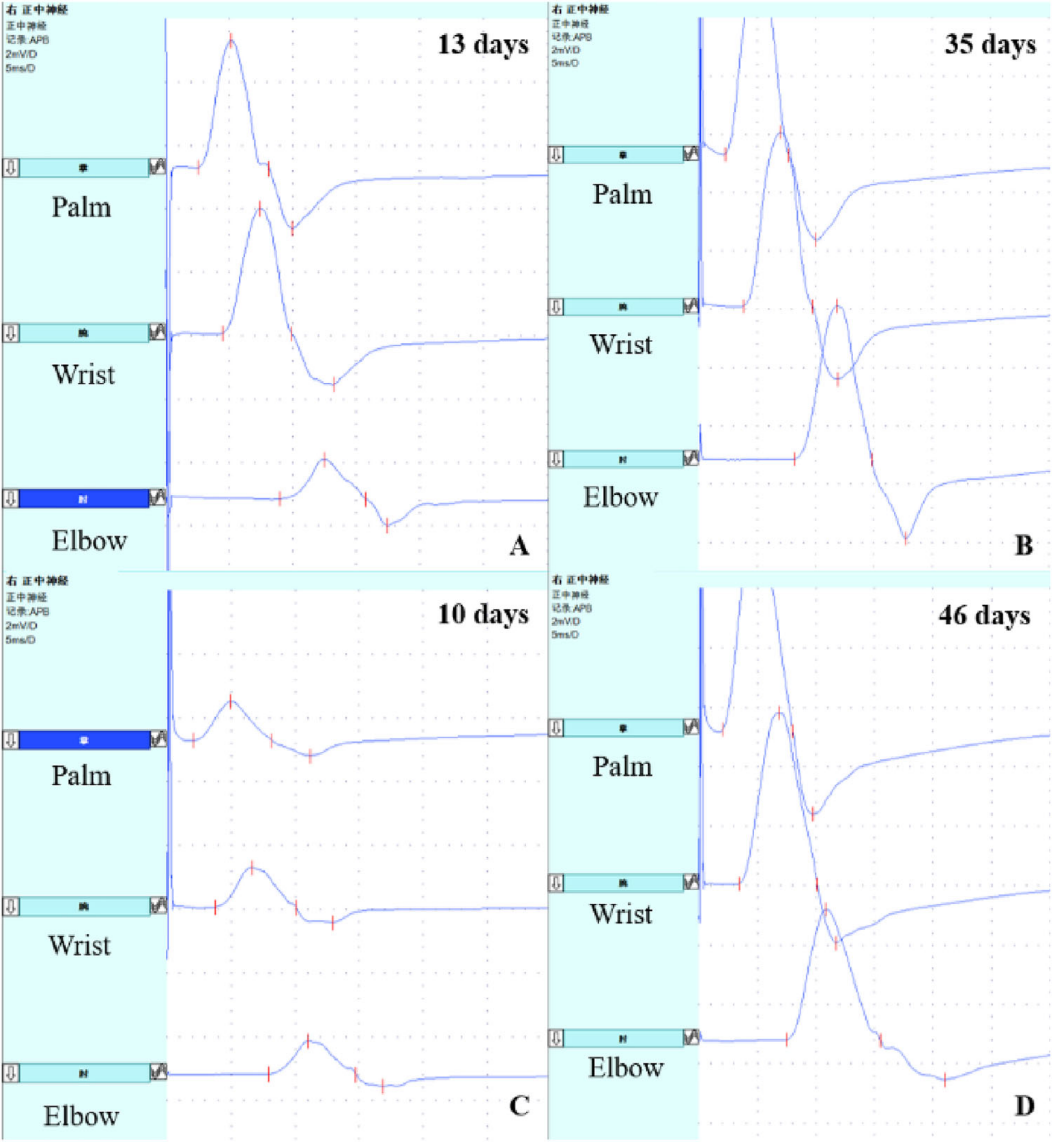
The initial and second electrophysiological findings in the median nerve of two RCF patients. Patient one was a 57‐year‐old male who underwent the initial study on Day 13, which revealed CB between the elbow and wrist (A). Patient two was a 59‐year‐old female who underwent the initial study on Day 10, which revealed slightly prolonged DML and low CMAP amplitude from the palm to the elbow without CB (C). Both patients recovered rapidly after intravenous immunoglobulin therapy, as shown in the second examinations conducted on Days 35 and 46, respectively (B and D). CB, conduction block; CMAP, compound muscle action potential; DML, distal motor latency; RCF, reversible conduction failure.

Figure 2 presents the initial and second electrophysiological findings in the median nerve of two RCF patients. One patient had CB (Figure 2A), whereas the other showed a slightly prolonged DML without CB (Figure 2C). Both patients recovered rapidly after intravenous immunoglobulin therapy during the second examination (Figure 2B,D).

## 4 Discussion

The present study investigated the initial clinical and electrophysiological characteristics of AIDP, axonal degeneration, and RCF subtypes of GBS diagnosed based on serial electrophysiological examinations. The different diagnostic classifications based on the initial and serial electrophysiological findings in our study indicated that early identification of RCF may be the greatest difficulty in the differential diagnosis of GBS. Therefore, we further explored the differences between RCF and AIDP and axonal degeneration to uncover clues for the early identification of RCF.

The differential diagnosis of RCF and AIDP is one of the difficulties. Our study indicated that AIDP patients usually had higher CSF protein than RCF patients. High CSF protein level has been reported to be associated with an early severe disease course and a demyelinating subtype (Al‐Hakem et al. 2023). The greater elevation of CSF protein in AIDP may originate from the more extensive blood–nerve barrier disruption and more intense inflammatory response (Al‐Hakem et al. 2023; Yuki and Hartung 2012). Although we could not establish an appropriate diagnostic cutoff due to a small sample size, we could see the overall trend of DML, *F*_min_, *F*_mean_, and *F*_max_ in RCF patients showed normal or slightly prolonged, rather than apparently prolonged in AIDP patients. Uncini et al. (2017) and Uncini and Kuwabara (2012) recorded in their articles that transient CB/slowing in intermediate and distal nerve segments, mimicking demyelination but without the development of abnormal temporal dispersion is an important feature of RCF. The slightly prolonged DML in RCF patients mostly came from conduction slowing in the distal segment. Moreover, the reduction of *F*% mainly resulted from conduction failure along the nerve. Because tibial nerve contains more motor axons than ulnar nerve, it is easy to elicit F‐waves and not easy to appear *F*% decrease for the former. In addition, the *F*% of the tibial nerve is higher than that of the ulnar nerve in normal people (Pan et al. 2013).

Differentiating between the RCF subtype and axonal degeneration subtype was another challenge in GBS diagnosis. Patients with RCF exhibited better motor function during hospitalization and had a favorable prognosis, as assessed by the Hughes functional grades at admission and discharge, as well as the variation from admission to discharge. In contrast, patients with axonal degeneration subtype typically experienced poor outcomes. This finding is consistent with the results reported in the study by Niu et al. (2018), where “AMAN with CBs” had a higher reduction of Hughes functional grade at 1 month than “AMAN without CBs.” The RCF and axonal degeneration groups shared similar electrophysiological features, except for CB, which was present in the former but not the latter. Due to the limited clinical and electrophysiological evidence available for distinguishing between RCF and axonal degeneration, early identification of RCF from axonal degeneration remains challenging, and further research is warranted.

The differential diagnosis between AIDP and axonal degeneration was relatively straightforward compared to distinguishing RCF from AIDP and axonal degeneration. Although demyelination is predominant in the former and axonal dysfunction in the latter, some patients exhibit both demyelination and axonal degeneration, making it challenging to differentiate (van Doorn et al. 2023). In comparison to axonal degeneration, the CSF protein levels of AIDP patients were significantly elevated. Additionally, prolonged DML and F‐wave latencies, along with decreased CMAP velocity, remained prominent features of AIDP.

Previous studies reported RCF was significantly associated with IgG antibodies to GM1, GalNAc‐GD1a, GD1b, or complex of LM1/GA1 (Créange et al. 2014; Ogawa et al. 2013; Shahrizaila et al. 2014). Antibodies bound to gangliosides at the nodes of Ranvier, which activated complement, disrupted sodium‐channel clusters, and resulted in “axonal CB.” As “axonal CB” brought confusion in the electrodiagnostic diagnosis of GBS, some researchers proposed the term “nodal CB” (Oh 2021). This kind of “CB” can promptly recover after immune‐modulated treatment without prominent pathological changes (Kaida 2019; Kuwabara and Yuki 2013). Conversely, if the block is not resolved quickly, they will develop axonal degeneration. Therefore, early identification of the RCF subtype can guide treatment in time. Some studies reported anti‐ganglioside antibodies play an important role in the different patterns of evolution, RCF, or axonal degeneration (Kuwabara, Asahina, et al. 1998). However, a study conducted by Kokubun et al. (2010) did not find a difference in anti‐ganglioside antibody profiles between patients with and without RCF. We also did not reveal a significant difference of ganglioside antibodies in CSF or blood among the three groups in our series.

In summary, we concluded the following points regarding three subtypes in the initial examinations. The early identification of RCF from AIDP had relatively obvious features, including slightly elevated CSF protein levels and normal or slightly prolonged DML and F‐wave latencies, rather than significantly elevated and prolonged as in AIDP. The early identification of RCF from axonal degeneration remains challenging. One differentiator that may be helpful was that the motor function of the RCF showed better than in the latter.

## Author Contributions

**Shuo Yang**: writing–original draft, methodology, formal analysis, data curation, writing–review and editing. **Na Chen**: methodology, data curation. **Lei Zhang**: methodology, data curation. **Ying Wang**: methodology, data curation. **Lin Chen**: data curation. **Fan Jian**: data curation. **Zaiqiang Zhang**: project administration, supervision. **Yilong Wang**: supervision, project administration. **Hua Pan**: writing–review and editing, supervision, validation, project administration.

## Ethics Statement

We confirm that we have read the Journal's position on issues involved in ethical publication and affirm that this report is consistent with those guidelines.

## Conflicts of Interest

The authors declare no conflicts of interest.

### Peer Review

The peer review history for this article is available at https://publons.com/publon/10.1002/brb3.70068.

## Data Availability

The data that support the findings of this study are available from the corresponding author upon reasonable request.
